# Major Cereal Grain Fibers and Psyllium in Relation to Cardiovascular Health

**DOI:** 10.3390/nu5051471

**Published:** 2013-04-29

**Authors:** Adam M. Bernstein, Brigid Titgemeier, Kristin Kirkpatrick, Mladen Golubic, Michael F. Roizen

**Affiliations:** Cleveland Clinic, Wellness Institute, 1950 Richmond Road/TR2-203, Lyndhurst, OH 44124, USA; E-Mails: titgembf@gmail.com (B.T.); kirkpak2@ccf.org (K.K.); golubim@ccf.org (M.G.); roizenm@ccf.org (M.F.R.)

**Keywords:** cereal, fiber, cardiovascular disease, heart disease, beta-glucan, psyllium, arabinoxylan, fructan, resistant starch

## Abstract

Numerous studies reveal the cardiovascular benefits of consuming dietary fiber and, especially, cereal fiber. Cereal fiber is associated with cardiovascular risk reduction through multiple mechanisms and consuming a variety of cereal fiber sources offers health benefits specific to the source. Certain cereal fibers have been studied more extensively than others and provide greater support for their incorporation into a healthful diet. β-glucan from oats or barley, or a combination of whole oats and barley, and soluble fiber from psyllium reduces the risk of coronary heart disease; inulin-type fructans added to foods and beverages may modestly decrease serum triacylglycerols; arabinoxylan and resistant starch may improve glycemic control. Individuals with low cereal fiber intake should increase their intake of whole grains in order to receive the benefits of whole grains in addition to fiber. For those adjusting to the texture and palatability of whole grains, turning to added-fiber products rich in β-glucan and psyllium may allow them to reach their fiber goals without increasing caloric intake.

## 1. Introduction

Ninety percent of the U.S. population does not consume enough dietary fiber [1]. Although currently there is not enough evidence to set a Recommended Dietary Allowance (RDA) for fiber intake [2], the Institute of Medicine (IOM) Adequate Intake (AI) guidelines recommend 14 g per 1000 kcal or about 25 g/day for women and 38 g/day for men [3]. However, because breads and pizza made with refined flour are eaten so often, the average American consumes only 15 g/day [4], and intake may be even less than 10 g/day for those on a low-carbohydrate diet [5]. This inadequate intake presents a major public health problem given that low intake is associated with an increased risk for cardiovascular disease (CVD).

The cardiovascular benefits of fiber and, especially, cereal fiber, have been well documented: cereal fiber is strongly associated with a reduced risk of myocardial infarction, total and ischemic stroke, and incident cardiovascular disease [6,7], as well as death from heart disease [7,8,9]. These benefits are likely achieved through multiple metabolic pathways [10,11,12]: by reducing weight and waist circumference [13], body mass index (BMI), percent body fat and percent trunk fat mass [14]; improving glucose metabolism and insulin sensitivity [15,16,17]; and lowering the risk of metabolic syndrome [18] and diabetes [19].

Consumption of whole grains confers the benefits of cereal fiber plus those from a wide range of other protective compounds, including vitamins, minerals, antioxidants (some of which are bound to fiber), phytosterols, unsaturated fatty acids, phytin, and lignans [20]. The relationship between whole grains and cardiovascular risk reduction has been well studied [20]. The association between individual types of cereal fiber and post-prandial satiety, energy and weight change has also been discussed [21]. What remains less clear is how individual types of cereal fiber relate to CVD risk. 

This review focuses on the relationship between major types of cereal fiber and CVD risk factors, such as elevated blood glucose and cholesterol. It also looks at psyllium in relation to CVD risk. The review allows not only for a synthesis of current evidence, but also for identifying gaps in knowledge, such as how various cereal fibers commonly added to foods compare in terms of established or potential cardiovascular benefit. It also presents the known benefits and properties of specific fiber types, and where they can be found, to help inform individuals’ dietary choices. 

## 2. Grain Components and Fiber Types

**Figure 1:**
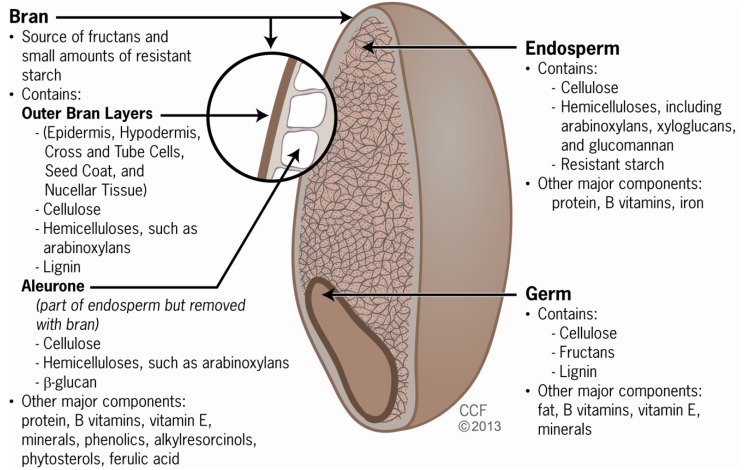
Fibers of the Wheat Kernel.

Cereals, also called grains or grain crops, are grasses from which the seed (also called the grain or kernel) is removed for consumption. Cereals commonly consumed are rice, wheat, maize (corn), barley, rye, oats, millet and sorghum; of these, barley and rye contain the most fiber per gram of edible portion; rice and millet the least [22]. The grain contains three major components—bran, germ and endosperm. Fiber is found in each component, although each component has a different composition of fiber types (example provided in Figure 1). Bran is included in whole wheat flour, but more often added to animal feed; germ is separated during the refining process because of its fat, which may oxidize; endosperm is the source of white flour [23]. The fiber-rich bran and germ include many micronutrients and phytochemicals, whereas the endosperm consists mainly of starch [24]. Protein is chiefly found in the endosperm and bran [23]. Fibers found in these parts of the grain have different physical and chemical characteristics, varying potential or well-studied cardiovascular benefits, and multiple contributions to the chemistry of food (Table 1).

Cellulose is a fiber often used as a placebo in clinical trials given that it has little apparent effect on blood lipids or glucose [3]. Hemicelluloses are a group of polysaccharide fibers associated with plant cell wall components, such as cellulose, proteins, lignins and phenolic compounds [25]. Hemicelluloses present in cereals are chiefly arabinoxylans, β-glucans, pectins, and arabinogalactans; the latter two constitute a small part of the grain and are not thought to contribute substantially to grain’s association with health benefits [26]. Xyloglucans and gluco- and galacto-mannans are also considered minor hemicelluloses [25]. The minor cereal grain fibers will not be reviewed here. Lignin is a compound typically considered a type of dietary fiber, but also will not be reviewed given that its cardiovascular effects have not been clearly defined. Fructans and resistant starch, two other grain fibers, will be reviewed given they have been extensively studied, and psyllium will be considered, given that it is commonly incorporated into grain-based foods and its chief component is arabinoxylan [27].

**Table 1:** Major dietary fibers from cereal grains with established or investigated cardiovascular health benefit ^1^.

Fiber	Water Soluble (S) or Insoluble (I)	Viscosity	Fermentability	Major Cereal Sources	Properties that May Assist with Incorporation into Food [28]	Cardiovascular Benefit [29]
Beta-glucans	S	Highly viscous	High	Oats, barley	Able to be added to a range of different products, including cereal, soup, beverages	≥3 g/day of β-glucan soluble fiber from whole oats or barley, or a combination thereof, can reduce the risk of coronary heart disease
Arabinoxylans ^2^	S	Viscous	High	Barley, wheat, rye, rice, sorghum, oats, corn millet	May lower glycemic index of breads, while providing pleasing mouth feel and tenderness	≥7 g/day of soluble fiber from psyllium seed husk can reduce the risk of coronary heart disease
Inulin-Type Fructans	S	Mostly viscous	High	Wheat	Used to replace fat or carbohydrates without affecting taste or texture	Not yet established
Resistant Starch ^3^	S	Non-viscous	Variable (rate and degree depend on source and heat treatment)	RS1: partially milled grainsRS3: cooked & cooled rice, pasta	Palatable and provides mouth feel of refined carbohydrates	Not yet established

^1^ Data from multiple sources [1,3,12,23,30,31,32,33,34,35,36,37,38,39,40,41,42,43,44]; ^2^ Major component of psyllium considered to be an arabinoxylan [27]; ^3^ RS1: Resistant Starch Type 1; RS3: Resistant Starch Type 3.

3. β-Glucan 

β-glucan, a soluble non-starch polysaccharide composed of linear chains of glucose, is commonly found in grains, such as oats and barley, which contain similar concentrations of the fiber [45]. Many studies have looked at the relationship between β-glucan and cardiovascular health (Table 2). One meta-analysis reported that 2–10 g/day of soluble fiber from oats (largely β-glucan [46]) resulted in small, but significant, decreases in total cholesterol (TC) (−0.04 mmol/L per g; 95% CI: −0.05, −0.03 mmol/L per g; equal to −1.55 mg/dL per g; 95% CI: −1.93, −1.16 mg/dL per g) and low-density lipoprotein cholesterol (LDL) (−0.04 mmol/L per g; 95% CI: −0.04, −0.03 mmol/L per g; equal to −1.55 mg/dL per g; 95% CI: −1.55, −1.16 mg/dL per g) [47] and that intake of 3 g per day of soluble fiber from three bowls (28 g servings each) of oatmeal could decrease TC by approximately 0.13 mmol/L (5.02 mg/dL) or 2% [47]. On a population level, this magnitude of reduction could lower the incidence of coronary disease by about 4% [47]. Individuals with particularly high TC levels (≥229 mg/dL) may see the greatest benefit in cholesterol reduction [48].

Another meta-analysis confirmed the significant inverse relation in TC (−0.60 mmol/L; 95% CI: −0.85, −0.34 mmol/L; equal to −23.2 mg/dL, 95% CI: −32.9, −13.1 mg/dL) and LDL (−0.66 mmol/L, 95% CI: −0.96, −0.36 mmol/L; equal to −25.5 mg/dL, 95% CI: −37.1, −13.9 mg/dL) and also reported an increase in high-density lipoprotein cholesterol (HDL) (0.03 mmol/L, 95% CI: −0.06, 0.13 mmol/L; equal to 1.16 mg/dL, 95% CI: −2.3, 5.03 mg/dL) after consumption of β-glucan from oats and barley and foods made from these grains [49]. The larger effect seen in this study compared to the earlier one may be due to different number of included studies, as well as differences in inclusion and exclusion criteria (the earlier study included only controlled studies; the latter included cohort, dose-response and pre-post treatment studies). The latter meta-analysis also showed a significant change in blood glucose (−2.58 mmol/L, 95% CI: −3.22 to −1.84 mmol/L; equal to −46.4 mg/dL, 95% CI: −57.8, −33.1 mg/dL) and reported that while β–glucan from oats and barley both decreased TC and LDL; only oats increased HDL. The study concluded that an intake of 3 g/day of β–glucan could produce a modest decrease in TC of −0.30 mmol/L (equal to 11.58 mg/dL), but that no significant additional change in TC would occur with intake above 3 g/day [49]. 

Two additional meta-analyses verified the TC and LDL lowering effects of whole grain barley and β-glucan from barley [45,50]. One noted that the processing of β-glucan (e.g., baking it into bread, adding it to cereal or soup, or blending it into beverages) may affect its cholesterol-lowering ability, because changes to the β-glucan structure and solubility can occur with processing and the lipid-lowering effects depend on the food matrix [50]. Nevertheless, the report stated that β-glucan from barley could lower TC and LDL whether it was incorporated into either a beverage or solid food [50].

Given the evidence for consuming oats, barley, and β-glucan intact in whole foods or added to foods as means to modestly lower total and LDL-cholesterol, U.S. federal regulations allow health claims to be made on foods stating that 3 g or more per day of β-glucan soluble fiber from either whole oats or barley, or a combination of whole oats and barley, can reduce the risk of coronary heart disease [29]. 

**Table 2:** Meta-analyses on β-glucans and psyllium in relation to serum cholesterol.

First Author (year)	Number of included studies (Number of participants) (*n*)	Age (mean or median) and/or age range of participants (years)	Intervention (mean, median, or range of dose)	Intervention duration (mean and/or range, days)	Main findings (mg/dL) (95% CI)	Risk of publication bias
Ripsin (1992) [48]	10 (1371)	20–73	Oat products (average soluble fiber dose range: 1.1–7.6 g/day)	18–84	TC: −5.9 (−8.4, −3.3)	Not reported
					Larger reductions observed with 3 g/day in participants with TC ≥ 229 mg/dL	
Brown (1999) [47]	25 (1600)	48 (26–61)	Oat products (average soluble fiber dose: 5 g/day with range of 1.5–13.0 g/day)	39 (14–84)	TC: −1.55 (−1.93, −1.16) per g	Not reported
					LDL: −1.55 (−1.55, −1.16) per g	
Talati (2009) [45]	8 (391)	Not reported	β-glucan from barley (7 g/day with range of 3–10 g/day)	28–84	TC: −13.38 (−18.46, −8.31)	Low
					LDL: −10.02 (−14.03, −6.00)	
					TG: −11.83 (−20.12, −3.55)	
AbuMweis (2010) [50]	11 (591)	20–63	Barley or β-glucan from barley (5 g/day)	28–84	TC: −11.60 (−15.08, −8.12)	Possible (asymmetric funnel plots)
					LDL: −10.44 (−13.15, −7.73)	
Tiwari (2011) [49]	20 (1154 (TC, LDL), 1000 (HDL))	18–72	β-glucan (2–14 g/day)	21–84	TC: −23.2 (−32.9, −13.1)	Indeterminate (low risk by Eggers test, but possible risk by funnel plot)
					LDL: −25.5(−37.1, −13.9)	
					HDL: 1.16 (−2.3, 5.03) with oat β-glucan	
					3 g/day of β-glucan sufficient to decrease TC by 11.58 mg/dL	
Olson (1997) [51]	12 (404)	27–72	Psyllium-enriched cereal products (average soluble fiber dose range: 3–12 g/day)	14–56	TC: −11.99 (−14.31, −9.67)	Not reported
					LDL: −13.53 (−15.47, −11.21)	
Brown (1999) [47]	17 (757)	51 (44–59)	Psyllium (average soluble fiber dose 9.1 g/day with range of 4.7–16.2 g/day)	53 (14–112)	TC: −1.5 (−1.93, −1.16) per g	Not reported
					LDL: −2.7 (−5.8, −0.5) per g	
Anderson (2000) [52]	8 (384)	55 (24–82)	Psyllium (10.2 g/day)	56–182	TC: −9.20 (−12.69, −5.71)	Not reported
					LDL: −10.87 (−14.04, −7.70)	
Wei (2009) [53]	21 (1717)	Not reported	Psyllium (3–20.4 g/day)	14–182	TC: −14.50 (−9.94, −19.10)	Possible
					LDL: −10.75 (−8.24, −12.06)	
					Dose response observed with 5, 10 and 15 g/day resulting in 5.6%, 9.0% and 12.5% decreases in LDL	

TC, total cholesterol; LDL, low-density lipoprotein cholesterol; HDL, high-density lipoprotein cholesterol.

## 4. Arabinoxylan

Arabinoxylan is a polysaccharide fiber with xylose backbone and arabinose side chains that constitutes nearly 70% of the non-starch polysaccharide in wheat bran and 90% in wheat endosperm [39]. Its intake has been associated with improved glycemic control; in one small study with 12 participants, adding 6 or 12 g of arabinoxylan to bread resulted in a post-prandial area under the curve for glucose that was 20% (95% CI: 5.8%, 34.7%) and 41% (25.9%, 56.8%) lower, respectively, than control 2 h after the meal [39]. These glucose and insulin responses were similar to those obtained in other studies with the fibers guar gum and psyllium [39]. Another study with 11 participants and 15 g/day of arabinoxylan added to bread and other foods (e.g., yogurt, applesauce, and juice) over six weeks led to significantly lower post-prandial glucose, insulin and triglycerides [54,55]. However, a study with 14 participants consuming 15 g/day showed that while post-prandial glucose and insulin were lower, there was no change over five weeks in blood lipids, body weight, fat mass, or blood pressure [56]. Additionally, arabinoxylan-oligosaccharide, a fiber produced by enzymatic manipulation of arabinoxylan that has been studied as a prebiotic [57,58], also did not have an effect on blood lipids after three weeks [57]. 

Thus, while it appears that the addition of arabinoxylan to food products may improve glycemic control, studies are limited by short duration and small sample sizes and health claims for cardiovascular benefit have not been established. 

However, the active component of psyllium, which has been the focus of many studies since it was reported to lower cholesterol in 1965 [59], is thought to be an arabinoxylan [27] (Table 2). Psyllium is not derived from a cereal crop, but from the husk of the seed of the *Plantago* plant, a broad, green, flowering plant grown in areas such as China, India, and the Mediterranean and is known for the mucilaginous properties of its seed husks [3,53]. One meta-analysis reported that 2–10 g/day of psyllium results in small, but significant decreases in TC (−0.04 mmol/L per g; 95% CI: −0.05, −0.03 mmol/L per g; equal to −1.55 mg/dL per g; 95% CI: −1.93, −1.16 mg/dL per g) and LDL (−0.7 mmol/L per g; 95% CI: −0.15, −0.01 mmol/L per g; equal to −2.7 mg/dL, 95% CI: −5.8, −0.5 mg/dL per g) [47]. Another meta-analysis found that adding 3 to 12 g/day in breakfast cereal results in lower TC (0.31 mmol/L (12 mg/dL) or 5%) and LDL (0.35 mmol/L (13.5 mg/dL) or 9%) [51]. A meta-analysis of psyllium among adults with hypercholesterolemia following a low-fat diet concluded that 10.2 g/day of psyllium lowers TC by 4% and LDL by 7% and reduces the ratio of apolipoprotein B to apolipoprotein A1 by 6% [52]. Consuming 5, 10, or 15 g/day was estimated to result in 5.6%, 9.0% or 12.5% lower LDL [53].

A more recent study identified additional CVD benefits of psyllium consumption: in men with coronary heart disease, psyllium significantly reduced triglycerides (TG) by 6.7%, increased HDL by the same amount, improved the apolipoprotein B to apolipoprotein A1 ratio by 4.7%, increased apo A-1 by 4.3%, and decreased waist circumference and the waist-to-hip ratio [60]. The apo B to apo A-1 ratio has been suggested to be an ideal marker of atherogenic and anti-atherogenic particles in plasma [60]. Psyllium has also been shown to lower blood pressure [61] and serum glucose [3]. 

With this range of cardiovascular health benefits, U.S. federal regulations allow health claims to be made on foods stating that 7 g or more per day of soluble fiber from psyllium seed husk can reduce the risk of coronary heart disease [29]. 

## 5. Resistant Starch and Fructans

Starch, found abundantly in the endosperm of the cereal grain, is composed of amylose, a linear glucose polymer, and amylopectin, a branched glucose polymer. In many foods, a small proportion of the starch is resistant to the usual digestive process (typically 0–5% of the starch in cereal products), although for some foods, such as legumes, this percent is higher (e.g., 10%–20% for some beans) [62]. As a result, this resistant starch behaves as dietary fiber. Four main subtypes of resistant starch have been identified based on their structure or source: that which is physically inaccessible to digestive enzymes is called *Resistant Starch type 1* (RS1) and is found in whole or partly milled grains and seeds and whole-grain foods; that which is resistant to digestion due to the nature of the starch granule is referred to as *Resistant Starch type 2* (RS2) and is found in raw potato, unripe banana, some legumes, and high-amylose corn; that which forms from retrograded amylose and amylopectin during food processing is called *Resistant Starch type 3* (RS3) and is found in cooked and cooled foods such as potatoes, bread, and cornflakes; *Resistant Starch type 4* (RS4) is produced by chemical modification [63]. As each type has its own physical and chemical properties that influence the rate and site of fermentation in the human gut, the properties associated with one type cannot necessarily be extrapolated to others [62].

Although there is limited long-term data on resistant starch’s effect on glucose metabolism [64], short-term evidence from multiple studies with small study populations suggests that RS2 from high-amylose corn can improve insulin sensitivity [65,66,67,68], even without changes in body weight, visceral or hepatic fat, or inflammation [67]. There is also a reduction in the blood sugar rise after a single meal, an effect seemingly enhanced by combining resistant starch with β-glucan [69]. A threshold of 5–6 g appears needed for reductions in the insulin response [69].

Like resistant starch, inulin-type fructans, including inulin and oligofructose (also called fructo-oligosaccharide) are components of cereal grains that are considered a type of dietary fiber whose effect on cardiovascular risk has not been firmly established. Fructans are defined as linear polydisperse carbohydrates consisting mainly, if not exclusively, of certain fructosyl-fructose linkages [70]. Wheat is a common dietary source of fructans, as well as onions, bananas, garlic, leeks, and Jerusalem artichokes. Commercially available forms of fructans are extracted and purified from chicory root or Jerusalem artichoke or are synthesized from sucrose [70,71].

A 2002 Institute of Medicine report concluded that the relationship between inulin and oligofructose on plasma lipids was uncertain [3]. A more recent meta-analysis of 15 controlled studies showed that these two compounds, at an average intake of 14 g/day, added to foods and beverages (including sweeteners, biscuits, yogurt, breakfast cereal, ice cream, orange juice, fermented milk, chocolate, and spreads) were associated with a significant decrease in serum triglycerides of −0.17 mmol/L (95% CI: −0.33, −0.01 mmol/L), equal to 15.0 mg/dL (95% CI: 29.2, 0.9 mg/dL) or 7.5% [72]. Inulin appears more effective than oligofructose at this function [70]. A systematic review appearing after the meta-analysis reported that in four out of 13 randomized controlled trials, dietary fructans decreased serum glucose concentration, although only one was statistically significant and nine showed no significant change [71]. Reasons for these inconsistent findings may be due to varying placebos, participant characteristics, fructan doses, and study duration [71].

Thus, while it appears that resistant starch may improve glycemic control and fructans may lower triglycerides, evidence is limited and health claims for cardiovascular benefit have not been established. 

## 6. Biological Mechanisms

Viscous, or gel-forming, fibers (pectins, gums, mucilages, and certain hemicelluloses, such as arabinoxylans and β-glucans) are generally soluble, while the structural or matrix fibers (lignins, cellulose, and certain hemicelluloses) are generally insoluble [47]. Soluble fibers appear to beneficially impact gut hormones, such as cholecystokinin [73], glucagon-like peptide 1, and peptide YY and ghrelin [74], which control satiety, and insoluble fibers have been linked with changes in glucose-dependent insulinotropic polypeptide, which is related to postprandial insulin secretion [75]. In addition, soluble fibers exert physiological effects on the stomach and small intestine by delaying the gastric emptying rate and small bowel transit time, increasing satiety, and slowing absorption of nutrients, such as glucose, triglycerides and cholesterol [30,52]. Soluble fiber also increases bile acid excretion from the liver, thus diverting hepatic cholesterol towards bile acid production [52]. Fermentation of fiber in the large intestine decreases cecal pH and increases bacterial biomass leading to an increase in fecal output and the production of gases (carbon dioxide, methane, hydrogen) and short-chain fatty acids (primarily acetate, propionate and butyrate) [3]. The short-chain fatty acids (SCFAs) act as the main energy source of gut epithelial cells [3], while also inhibiting hepatic cholesterol synthesis [52], and decreasing free fatty acid and glycerol release from adipose tissue, a reduction which in turn can enhance insulin sensitivity [65].

Resistant starch and inulin-like fructans lack viscosity, but are fermented in the colon to SCFAs [76]. Resistant starch is thought to decrease satiety by increasing levels of gut hormones, such as glucagon-like peptide-1 or peptide YY [77]. Although inulin-type fructans can modulate gastrointestinal microflora with the selective stimulation in growth of reported health-promoting bacteria (e.g., *Bifidobacterium*) [70], their ability to reduce triglycerides is likely mediated by downregulation of hepatic lipogenesis [72].

## 7. Public Health Fiber Recommendations and Implications

Recent estimates are that 36% of the U.S. population falls below the minimum recommended intake for grains (between 84 and 224 g/day, depending on age and gender) and 99% falls below the recommendation for whole grains (42 to 112 g/day, depending on age and gender) [78]. The U.S. Department of Agriculture recommends increased consumption of whole grains, as well as beans, peas and vegetables, fruits and other foods with naturally occurring fiber, to help increase fiber intake [4]. Following these recommendations, one would get two-thirds of daily fiber from fruits and vegetables and one-third from grains, of which the majority would be from whole grains [1]. These guidelines follow upon evidence that the total amount of dietary fiber, rather than that from any particular source, is most important to health [79]. 

In addition to incorporating more whole grains into the diet, replacing refined grain products with added-fiber, grain-based foods may allow individuals to increase their fiber intake [1,80]. Such a substitution should not substantially increase caloric intake. For example, one slice of whole grain bread (1.9 g of fiber) has 69 Kcal and one slice of white bread (0.7 g fiber) has 66 kcal [81]; fiber yields at most 2.4 kcal/g, as opposed to carbohydrate absorbed in the small intestine, which yields 4 kcal/g [82]. Thus, adding fiber to refined grain products, or substituting it for carbohydrate, may not add substantially to the caloric content of the food. Consuming fiber-added foods allows for an increase in fiber intake, much as foods fortified with folate or vitamins D allow for increased intake of these nutrients [1]. Manufacturers are currently developing products with ingredients that offer the nutrition of whole grain, but with a taste and texture similar to that of refined grains [83]. However, and importantly, added-fiber products may not confer all the benefits of whole grains, which contain fiber plus a wide range of other healthful compounds, including vitamins, minerals, phytosterols, unsaturated fatty acids, phytin, lignans, and antioxidants [20]. 

There appear few adverse effects of increasing cereal fiber or psyllium intake [3]. The most common possible adverse effects include intestinal gas and bowel discomfort; decreased absorption of zinc, calcium, and iron; and decreased intestinal transit time [3,84,85,86]. In one study of patients with a history of colorectal adenoma, psyllium intake was also associated with an increased risk of adenoma recurrence, especially in those patients with a high dietary calcium intake [87]. Intestinal gas production depends on the rate and amount of fiber and fluid intake, as well as the fermentability of the particular fiber [88]. Fiber supplements, such as Metamucil, made of psyllium, should be taken with 8 oz of water to prevent choking and at least 2 h apart from oral medications to prevent possible interaction [3]. There is no clear evidence of mineral deficiency associated with high fiber intake and, in fact, changes in absorption may be due not to fiber, but to the chelation capacity of phytate associated with certain fibers [3,40]. 

Although an upper limit on the amount of fiber that can be consumed has not been established, excess consumption is likely to be self-limiting due to fiber’s bulky nature [3]. In one study, consuming up to 59 g/day of fiber from food and supplements appeared safe [89]. The risk of adverse effects from overconsumption can likely be decreased by slowly adding fiber to the diet and maintaining adequate fluid intake.

## 8. Conclusions

Cereal fiber, as a heterogeneous nutrient category, is associated with a host of health benefits: reductions in weight and waist circumference [13], body mass index, percent body fat and percent trunk fat mass [14]; improvements in glucose metabolism and insulin sensitivity [15,16,17]; decreased risk of metabolic syndrome [18] and diabetes [19]; and reduced risk of cardiovascular disease [6,7] and death [7,8,9]. However, certain individual types of cereal fiber have been studied more extensively than others in relation to cardiovascular risk reduction. It appears well-established that 3 g or more per day of β-glucan from oats or barley or 7 g or more per day of soluble fiber from psyllium can reduce the risk of coronary heart disease [29]. The relationship between other cereal fibers and cardiovascular health is less clear: 14 g/day of inulin-type fructans, added to foods and beverages, may modestly decrease serum triacylglycerols [72] and 6 g/day of arabinoxylan or 5–6 g/day of resistant starch may improve glycemic control [65]. Current evidence suggests that individuals who consume little cereal fiber would benefit from increasing their intake of whole grain foods high in fiber. For those individuals adjusting to the texture and palatability of whole grains, turning to added-fiber products rich in the well-studied β-glucan and psyllium may allow them to reach their fiber goals without increasing caloric intake. 
